# Prevalence of Combined Lipid Abnormalities in Brazilian Adolescents and Its Association with Nutritional Status: Data from the Erica Study

**DOI:** 10.5334/gh.769

**Published:** 2020-03-18

**Authors:** Tatiana L. Kaestner, Jamylle A. D. Santos, Daiane C. Pazin, Cristina P. Baena, Marcia Olandoski, Gabriela A. Abreu, Maria Cristina C. Kuschnir, Katia V. Bloch, Jose R. Faria-Neto

**Affiliations:** 1 Pontificial Catholic University of Parana (PUCPR), Curitiba, BR; 2 Federal University of Rio de Janeiro (UFRJ), Rio De Janeiro, BR; 3 State University of Rio de Janeiro (UERJ), Rio De Janeiro, BR

**Keywords:** dyslipidemia, adolescents, risk factors, screening

## Abstract

**Background:**

Cardiovascular diseases are the leading cause of death in Brazil and worldwide. The growing incidence of obesity in children and adolescents and its association with lipid abnormalities may worsen this scenario, mainly in developing countries where obesity has reached epidemic levels. Dyslipidemias have several patterns, and the combination of some lipid abnormalities may have higher atherogenic potential.

**Objectives:**

To evaluate the prevalence of single or multiple combined lipid abnormalities in adolescents and its association with nutritional status assessed by body mass index.

**Methods:**

Data were obtained from the Study of Cardiovascular Risks in Adolescents (ERICA), a school-based, national representative study with Brazilian adolescents between 12 and 17 years of age. Adolescents whose lipid profiles were available were included, and lipid abnormalities were defined as LDL-C ≥ 100 mg/dL, HDL-C < 45 mg/dL, and tryglicerides (TG) ≥ 100 mg/dL. We assessed the prevalence of single or combined lipid abnormalities and correlated this nutritional status with body mass index of low weight, normal, overweight, and obesity.

**Results:**

A total of 38,069 adolescents were included, with more than 24,000 of them presenting at least one lipid abnormality (64.7%), and 3.7% showing alterations in all of them. The most prevalent combination was high TG with low HDL-C levels. The higher the BMI, the more lipid abnormalities were found.

**Conclusions:**

In this large and representative sample of Brazilian adolescents, the majority had at least one lipid abnormality. Higher BMI was associated with a higher prevalence of combined lipid abnormalities.

**Highlights:**

– There is a high prevalence of Brazilian adolescents with dyslipidemias.
– BMI was associated with a higher prevalence of combined lipid abnormalities.
– BMI can be considered as an indicator of the diagnosis of dyslipidemia in adolescents.

## Introduction

Cardiovascular diseases (CVD), especially those of atherosclerotic origin, are the leading cause of death worldwide [1]. Although CVD commonly manifest in adulthood, risk factors and behaviors begin in childhood and adolescence, and early control delays the progression of the disease [2]. Among these factors, obesity is of particular interest because of its high prevalence, now increasingly affecting more individuals in their early years [3]. In the last few decades, there has been an increase in its prevalence, almost doubling since 1980, and becoming an important public health issue [4], mainly in developing countries [5].

In addition to being highly prevalent, obesity is associated with several chronic comorbidities that also increase cardiovascular risk by inducing lipid changes. Even in adolescents, there is a direct relationship between increased body mass index (BMI) and the appearance of lipid changes. A study with American adolescents found the prevalence of lipid alterations was 20.3%, reaching 42.9% in those who were obese [6]. The usual pattern of obesity-associated dyslipidemia is characterized by elevated triglycerides (TG) and low levels of high-density lipoprotein cholesterol (HDL-C), which results in an elevation of non-HDL cholesterol (nHDL-C). This pattern, called ‘combined dyslipidemia’ or ‘atherogenic dyslipidemia’, has been observed in more than 40% of obese American adolescents [6]. It is associated with pathological changes in atherosclerosis and vascular dysfunction in adolescence and young adults and predicts early cardiovascular events in adult life [7]. On the other hand, obesity is not necessarily associated with increased low-density lipoprotein cholesterol (LDLc) [8]. In adolescence, significant elevations of LDLc are infrequent [9] and, when they occur, they may be associated with familial hypercholesterolemia (FH) [10].

The development of public policies aimed to improve cardiovascular health in developing countries depends on understanding the updated scenario of risk factor prevalence. Since primordial and primary prevention targets younger ages, the ERICA study was designed to assess the prevalence of the main cardiovascular risk factors in Brazilian adolescents aged 12–17 years [11]. This was a pioneer study in a large and nationally representative sample. We have previously described the prevalence of dyslipidemias in different regions of Brazil [12] but not how various lipid abnormalities can coexist, and their association with nutritional status, which is the aim of this study.

## Methods

This study was conducted as part of the ERICA Study, a nationwide, school-based, cross-sectional study conducted in 2013–2014 that evaluated the prevalence of diabetes mellitus, obesity, hypertension, lipid abnormalities, insulin resistance, and inflammatory markers in adolescents aged 12–17 years who attended school in cities with >100,000 inhabitants in Brazil [11,12].

The sample had been stratified (27 capital cities and five strata composed of cities with >100,000 inhabitants from each of the five country regions) and conglomerated (by school and grade level) at the sample selection stage. Sample weight was calculated using the product of inverse inclusion probabilities in each sampling stage and was calibrated by considering the projected number of adolescents enrolled in schools located in the geographical strata, on December 31, 2013. A pre-stratification estimator was used for modifying the natural weight of the design through a calibration factor that corresponded to the ratio of the total population to the total estimate by using the natural post-stratification weight or estimated domain of the design, more details on the sample design can be found in Vasconcellos et al. [13].

All the students were interviewed and then submitted a consent form signed by them and their parents/guardians. The following exclusion criteria were applied: students not aged 12–17 years, pregnant adolescents, and individuals with mental or physical disabilities that could prevent evaluations and anthropometric measurements.

### Data collection

A standardized research protocol was adopted for blood collection and used in 27 centers (27 states) [11]. All the participating laboratories received the protocol documentation for all the steps, from scheduling to transportation to the central unit. The adolescents were instructed to fast for 12 hours before sample collection and completed a questionnaire for compliance confirmation. A single reference laboratory (central unit) was used for all the biochemical analyses: triglycerides (TG), HDL-C, glucose, glycated hemoglobin, fasting insulin, and total count levels were evaluated [14]. The LDLc level was calculated using the Friedewald equation [15].

### Analysis of combined lipid changes

In this analysis, we evaluated the combination of lipid changes: LDLc ≥ 100 mg/dL, HDL-C < 45 mg/dL, and TG ≥ 100 mg/dL.

Adolescents healthy into eight groups: profile 1, adolescents with normal lipid profile variables; profiles 2, 3 and 4, those with only one altered variable; profiles 5, 6 and 7, those with two altered variables; and profile 8, participants with all variables of the lipid profile altered. The prevalence of each profile was estimated for each category of nutritional status.

Adolescents with a BMI z-score for their sex and age group that was ≤–2 were considered to have very low or low weight; those with z-scores between >–2 and <+1 were considered to have an appropriate weight; when the z-score was ≥+1 and <+2, they were classified as being overweight; and with a z-score ≥+2 they were obese [16].

### Statistical analyses

Prevalence and 95% confidence interval (CI) were calculated for each lipid according to sex, age, school category, countrywide, and region. Mean values were estimated for quantitative variables, and proportions were calculated for qualitative variables. A 95% CI was adopted for both cases.

Characteristics of the study population were adjusted according to the sample design, using statistical routines for complex samples. For comparison of ages, the Poisson model was used to calculate the prevalence rate ratio (PRR) using 12 years of age as reference. Analyses were performed by using Stata 14.0v software.

### Ethical aspects

The study was approved by the research ethics committees of the Central Institute for Research Coordination (IESC/UFRJ) and the Pontifical Catholic University of the State of Paraná (PUCPR).

## Results

Data were analyzed from 38,069 adolescents who answered the questionnaire and had laboratory parameters of interest. About two-thirds were female, and most of them were in public schools (Table 1). A separate analysis of each lipid profile variable was previously published [12].

**Table 1:** Sample distribution by gender, age range, type of school (public or private), geographical region and nutricional status.

Variable	Sample	%

General	38,069	100
**Gender**		
Male	15,247	40.0
Female	22,822	60.0
**Age**		
12–14 years	17,434	45.8
15–17 years	20,635	54.2
**Gender and age**		
Male, 12–14 years	7,140	18.8
Male, 15–17 years	8,107	21.3
Female, 12–14 years	10,294	27.0
Female, 15–17 years	12,528	32.9
**School**		
Public	28,167	74.0
Private	9,902	26.0
**Region**		
North	7,322	19.2
Northeast	11,821	31.1
Southeast	8,653	22.7
South	4,758	12.5
Midwest	5,515	14.5
**Nutricional Status**		
Overweight	6,635	17.5
Obese	3,097	8.2

**Figure 1:**
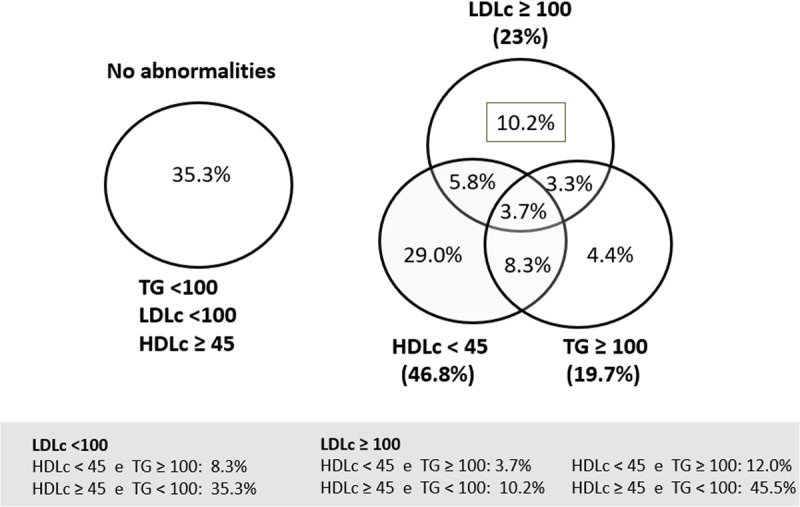
Combination of altered lipid variables among Brazilian adolescents.

More than 24,000 adolescents presented at least one altered lipid profile variable (64.7%), and 3.7% presented alterations in all variables (Figure 1).

The most commonly found lipid combinations were high TG and low HDL, and adolescents with this combination of lipid abnormalities had a greater mean of BMI-Z score (Table 2).

**Table 2:** Distribution of lipid variables with combined changes in 8 different profiles in increasing order of BMI z-score.

Profile	Triglyceride Desirable < 100 Borderline/high ≥ 100	LDLc Desirable < 100 Borderline/high ≥ 100	HDL-C Desirable ≥ 45	%	BMI z-score (mean, 95%)

1	Desirable	Desirable	Desirable	35.3	–0.06 (–0.11–0.01)
2	Desirable	**Borderline/high**	Desirable	10.2	0.24 (0.15–0.33)
3	**Borderline/high**	Desirable	Desirable	4.4	0.27 (0.12–0.42)
4	Desirable	Desirable	**Low**	29.0	0.29 (0.24–0.34)
5	**Borderline/high**	**Borderline/high**	Desirable	3.3	0.60 (0.44–0.76)
6	Desirable	**Borderline/high**	**Low**	5.8	0.69 (0.54–0.84)
7	**Borderline/high**	Desirable	**Low**	8.3	0.88 (0.78–0.98)
8	**Borderline/high**	**Borderline/high**	**Low**	3.7	1.36 (1.20–1.51)

**Graph 1:**
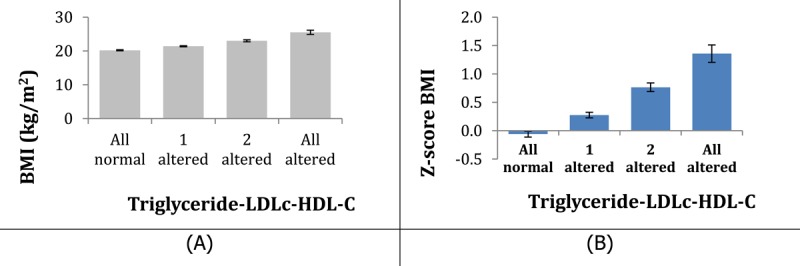
Relation of BMI **(A)** and z-score IMC **(B)** with number of altered lipid variables.

There was an association between the nutritional status and the number of lipid changes found: the higher the BMI, the more altered lipid parameters were found (Graph 1).

## Discussion

The contribution of developing countries to the global burden of cardiovascular diseases is significantly higher than that of the most developed countries [17]. The lack of awareness of lipid abnormalities and their associated factors is a barrier for achieving cardiovascular risk reduction [18]. In this study, we demonstrated that almost 65% of Brazilian adolescents have at least one lipid parameter deviating from the desirable range. Moreover, the number of lipid abnormalities increases together with an increase in BMI.

The recognition of risk factors in children and youth is critical for the development of prevention strategies. The excess of weight in the early years of life is predictive of a worse cardiovascular profile in adulthood. In the Bogalusa Heart Study, 2617 children and adolescents at ages 2 to 17 years were evaluated and lately were reexamined at ages 18 to 37 years (mean follow-up 17 years). In those with a BMI above the 95th percentile, 77% remained obese in adulthood, and this obesity was associated with abnormalities in lipids, blood pressure and insulin levels [19]. These findings reinforce the need for primordial and primary prevention.

Combined evidence from autopsy studies and cohort studies strongly indicate that abnormal lipid levels in childhood are associated with increased atherosclerosis [19]. Atherosclerosis, therefore, begins in youth and is related, from its earliest stages to the presence and intensity of known cardiovascular risk factors, such as family history, age, gender, diet, sedentary lifestyle, smoking, hypertension, dyslipidemia, overweight and obesity, diabetes and metabolic syndrome. Early identification and control of dyslipidemia throughout youth substantially reduces the risk of CVD in early adulthood. In a 25-year follow-up, the presence of risk factors of metabolic syndrome in childhood was associated with clinical CVD in adults between 30 and 48 years of age [8].

In our study, the majority of adolescents presented at least one altered variable of the lipid profile, which is in agreement with data reported in other populations. In 2011 and 2012, approximately 1 in 5 North American children and adolescents aged 8–17 years had adverse levels of the lipid parameters, total cholesterol, HDL-C, or non-HDL-C [20].

Among adolescents with two altered lipid variables, the altered variables most commonly associated with a high BMI z-score were high TG and low HDL-C. The Princeton Follow-up Study found that high TG and TG/HDL-C ratio for an average age of 12 years were predictors of cardiovascular disease 30–40 years later. It has been demonstrated that the TG/HDL-C ratio is the strongest predictor of the extent of coronary disease in adults, and is considered an alternative atherogenicity index of the plasma lipid profile [21].

It is also known that high BMI is related to risk factors and cardiovascular morbidity. In the United States, the Centers for Disease Control and Prevention reviewed the results of the National Health and Nutrition Examination Survey (NHANES) from 1999–2006. Of all the participants, 32% had a high BMI and were candidates for lipid screening under recommendations of the American Pediatric Association. There was a high prevalence of abnormal lipid levels among children and adolescent who were overweight (22.3%) or obese (42.9%) [20], as we have also demonstrated in our study.

The optimal way to screen for dyslipidemia in children and adolescents is not known. The recommendation from the National Cholesterol Education Program (NCEP) of the National Heart, Lung, and Blood Institute is that screening be made in children with a family history of early cardiovascular disease or high cholesterol. They also recommend screening for other risk factors in children with obesity, hypertension, and diabetes mellitus [8]. The United States Preventive Services Task Force stated in 2016 that there is insufficient evidence to assess the effect of screening for dyslipidemia in adolescents [22]. However, our study suggests that elevated BMI alone can be indicative of the need for dyslipidemia screening.

These results can be used not only to guide adolescents and parents about preventive measures but also to support the development of effective public health policies for the prevention and control of risk factors for diabetes and cardiovascular diseases in adolescents [11].

Strategies described to change this scenario among adolescents include lifestyle interventions [12], combined approaches to diet and physical activity for weight loss [23]; pharmaceutical treatment is rarely required [24], but always after an attempt of lifestyle changes [7]. Furthermore, some multidisciplinary pediatric programs seem to be more successful than programs for adults in maintaining weight loss for 5 or 10 years [23]. Recently the Familia study demonstrated that a four-month educational program with a multicomponent intervention promoting a healthy diet, increasing physical activity, understanding the human body, and managing emotions is an effective strategy to change behavior in children [25]. In Brazil, the Children First Study demonstrated that an educational program in cardiovascular prevention aimed at children aged 6 to 10 years can reduce the Framigham cardiovascular risk of their parents [26].

A comprehensive primary health care strategy that includes primary and secondary prevention and care and follow-up of cardiovascular diseases is associated with the reduction of morbidity and mortality due to cardiovascular diseases in Brazil [27], both in childhood and adulthood.

According to the World Health Organization, governments, the private sector, and civil society can work together to implement interventions on this issue. If they do, the overall goal for chronic disease prevention and control will be met, and millions of lives may be saved [1].

## Conclusions

Abnormal BMI of overweight/obesity in Brazilian adolescents directly reflects the alteration of the lipid profile.

We have also shown that the most common association between two altered lipid variables is that of TG and HDL-C together, representing a possible risk to Brazilian adolescents.

From this study, the use of BMI can be considered as an indicator of the diagnosis of dyslipidemia in adolescents, aiming at early interventions in the Brazilian lifestyle and consequent reduction of morbidity and mortality.
